# Dietary inflammatory index and odds of breast cancer: A case–control study

**DOI:** 10.1002/fsn3.2493

**Published:** 2021-07-28

**Authors:** Shatha S. Hammad, Reema Mahmoud, Nitin Shivappa, James R. Hebert, Lina Marie, Reema F. Tayyem

**Affiliations:** ^1^ Department of Nutrition and Food Technology Faculty of Agriculture The University of Jordan Amman Jordan; ^2^ Department of Epidemiology and Biostatistics University of South Carolina Columbia SC USA; ^3^ Cancer Prevention and Control Program University of South Carolina Columbia SC USA; ^4^ Connecting Health Innovations, LLC Columbia SC USA; ^5^ King Hussein Cancer Center Amman Jordan; ^6^ Department of Human Nutrition College of Health Sciences Qatar University Doha Qatar

**Keywords:** body mass index, breast cancer, dietary inflammatory index, proinflammatory, risk factors

## Abstract

Breast cancer (BrCA) is one of the most commonly diagnosed cancers and is the leading cause of cancer deaths in women worldwide. This study aimed to examine the association between the dietary inflammatory index (DII^®^) and BrCA among Jordanian women. A total of 400 adult women were enrolled into this case–control study. Cases were 200 women recently diagnosed with BrCA selected from the two hospitals that provide cancer therapy in Jordan. They were matched on age, income, and marital status with 200 BrCA‐free controls. DII scores were calculated from dietary data that were collected in a face‐to‐face interview conducted between October 2016 and September 2017 using a validated food frequency questionnaire. Conditional logistic regression models were used to calculate odds ratios (ORs) and 95% CIs. The study results revealed no significant associations between DII scores in relation to the odds of developing BrCA after multivariable adjustment including age, education, total energy, BMI, number of pregnancy, contraceptive use, lactation, smoking, and family history of BrCA. Stratified analyses by obesity status showed that overweight/obese participants in the highest DII tertile had a >75% increased BrCA risk (OR of 1.77 [95% CI, 1.01–3.12]) compared with participants in the lowest tertile, after adjusting for age. The results from this study showed no significant relationship between the proinflammatory potential of the diet and BrCA risk in the overall study population. However, results stratified by weight category indicated an effect of diet‐associated inflammation on BrCA risk in the overweight/obese group. Results of the study are consistent with a recommendation aimed at maintaining higher diet quality, that is, adopting healthy diets characterized by low DII scores in order to reduce the risk for BrCA.

## Graphical abstract

No significant relationship between the pro‐inflammatory potential of the diet and BrCA risk was detected among the study sample.

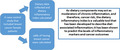

## 1 INTRODUCTION

Breast cancer (BrCA) is a generally indolent, but very common cancer that accounts for 20.8% of all newly diagnosed cancer cases of both genders and 37.4% of cancer cases among females according to the Jordanian National Cancer Registry (Ministry of Health, N.‐C. D. D., 2014). Even though it is a relatively indolent cancer, high incidence leads to it accounting for 10% of all cancer deaths (Ministry of Health, N.‐C. D. D., 2014).

Inflammation is a normal part of the normal biological repertoire needed for competent immune response to injury, infection, or other inflammatory stimulants in order to heal wounds, combat infections, detect early cancers, and promote tissue regeneration (Shivappa et al., 2015; Warnberg et al., 2009). However, tumor initiation, growth, and invasion can be triggered by a chronic, low‐grade inflammatory state that can be induced via an inflammatory microenvironment, which includes production of cytokines and chemokines (Cavicchia et al., 2009; Shivappa et al., 2015). Diet might be a key component in the regulation of chronic inflammation (Cavicchia et al., 2009; Shivappa et al., 2015), where the available evidence suggests the ability of dietary factors to influence the risk of BrCA through the modulation of inflammatory state (Research, A. I. f. C., 2007; Shivappa et al., 2015).

Certain culinary traditions, such as Mediterranean diets, which are rich in fruits, vegetable, healthy oils, fiber, β‐carotene, vitamin E, and vitamin C, have been recognized for their inverse association with chronic inflammation (Cavicchia et al., 2009; Shivappa, Blair, et al., 2017; Shivappa et al., 2016). On the other hand, the opposite is true regarding unhealthier diets, that is, typical Western‐style diets, that are high in fats, protein, simple carbohydrates, and refined carbohydrates but low in flavonoids and other antioxidant dietary components (Cavicchia et al., 2009; Shivappa, Blair, et al., 2017; Shivappa et al., 2016). Additionally, unhealthy diet also may lead to obesity, where the latter has been associated with increased production of estrogen, adipokines, and markers of inflammation. Obesity, as indicated by an increased body mass index (which is a rough proxy for adiposity), has been found to be associated with a significant direct relationship with risk of BrCA and an inverse association with survival after BrCA (Ferrini et al., 2015).

Because specific dietary components may act as moderators of chronic inflammation, and therefore, cancer risk, the dietary inflammatory index (DII^®^), is a valuable tool that has been developed to describe diet‐associated inflammation; it has been shown to predict the levels of inflammatory markers and cancer outcomes (Shivappa et al., 2014). The DII scores dietary components according to their potential inflammatory effects on a scale ranging from maximally anti‐inflammatory to maximally proinflammatory which will help examine the potential of a diet to induce cancer through modulating the inflammatory state. Although a large body of evidence suggests a central role of dietary factors in the development of BrCA, the relationship remains controversial with insufficient evidence to infer a probable causal association (Albuquerque et al., 2014; Ferrini et al., 2015; Shivappa, Blair, et al., 2017; Shivappa et al., 2015). To date, prospective epidemiologic studies failed to provide a significant, strong, reproducible association between diet and BrCA incidence (Ferrini et al., 2015). Here, we examined the association between the DII and BrCA among Jordanian women.

## 2 SUBJECTS AND METHODS

### 2.1 Study design and setting

This case–control study was conducted between October 2016 and September 2017. Its goal was to investigate the association between DII scores and BrCA among Jordanian women. This study was carried out at the main two hospitals in Jordan that offer cancer therapy: King Hussein Cancer Center (KHCC) and Al‐Basheer Hospitals. Permission was obtained from each hospital for having a private room in good physical condition that we could use to carry out the interviews was obtained.

### 2.2 Sample enrollment

Two hundred recently diagnosed (up to 3 months from diagnosis) BrCA patients were recruited from the Oncology department at KHCC and Al‐Basheer Hospitals. Patients were included based on the following criteria: recently (≤3 months) diagnosed with primary BrCA, Jordanian nationality, aged ≥20 years, and able to communicate verbally in Arabic. The exclusion criteria were hospitalized or critically ill, suffering from or previously diagnosed with other types of cancer and other diseases requiring a specific diet, and being pregnant or lactating. The control group was enrolled from the community of the participating hospitals including the employees and visitors, as well as patients' accompanying persons, none of whom could be a first‐degree relative. The assignment of participants into the control group was contingent on the performance of a mammogram or clinical examination, during the previous year, to ensure that they were free of BrCA. Cases and controls were recruited at a 1:1 ratio. Matching between cases and control groups was performed based on age as well as economic and marital statuses. Recruitment was accomplished via representatives from each participating hospital who called each patient to invite her to participate in the research. During the first interview, the investigator explained to participants the purpose and requirements of the study and elucidated potential risks and benefits knowing that the present study has no risk. The investigator also clarified that the participants can withdraw from the study at any time they want without any consequences.

### 2.3 Ethics

The protocol of this study was conformed to the ethical standards of the responsible committee on human experimentation and in compliance with the Helsinki Declaration of 1975, as revised in 1983. The Institutional Review Boards of the participating hospitals had reviewed and approved the proposal. Written informed consent form was completed and obtained before starting data collection from all participants where each participant was encouraged to read the consent form before signing it. Patients' information was kept and treated confidentially.

### 2.4 Data collection

A personal and demographic information sheet and a validated Arabic food frequency questionnaire (FFQ) (Tayyem et al., 2014) were used for data collection via a face‐to‐face interview by a trained researcher.

#### 2.4.1 Personal information sheet

This questionnaire included information on sociodemographic questions related to age, marital status, education, employment, family income, family members diagnosed with cancer, smoking status, medication, previous and current health problems, among others.

#### 2.4.2 Dietary assessment

The validated Arabic quantitative FFQ, which includes 109 questions on food and beverages, was used to collect dietary data. This FFQ was adapted from the Diet History Questionnaire of the US National Cancer Institute and was designed to measure the diet of Jordanians. The one‐year reference period, which was selected to reflect seasonal variation in some food types, aimed to cover the year before the diagnosis date for cases and the year before interview for controls. The average rate of consumption of each food item they had consumed at least one standard serving size was determined based on the following categories: “<1/month, 2–3/month, 1–2/week, 3–4/week, 5–6/week, 1/day, 2–3/day, 4–5/day, or 6/day.” Estimating the consumed portion size was facilitated via standardized food models (Nasco Company) and standard measuring tools. Food lists in the modified FFQ questions were classified based on types of foods: 21 items of vegetables; 16 items meat such as red meat (lamb and beef), chicken, fish, cold meat, and others; 21 items of fruits and juices; 9 items of milk and dairy products; 8 items of cereals; 4 items beans; 4 items of soups and sauces; 5 items drinks; 9 items of snacks and sweets; and 14 items of herbs and spices. Dietary analysis software (ESHA Food Processor SQL, version 10.1.1; ESHA) was used to analyze the dietary intakes.

#### 2.4.3 Physical activity level assessment

Weekly physical activity level of each participant was evaluated using the Sallis et al. (1985) physical activity recall (PAR) questionnaire (Sallis et al., 1985). The frequency, intensity, time, and type of physical activity were all considered. Thereafter, total physical activity was estimated using a metabolic equivalent score.

### 2.5 Anthropometric measurements

Measurement was taken according to Lee and Nieman (2012) (Lee & Nieman, 2012). Body weight (to the nearest 0.1 kg) was measured with the participant in bare feet and with minimal clothing, using a calibrated portable scale. Standing height was measured, using a calibrated portable measuring‐bar, to the nearest 0.5 cm where participants were in the full standing position without shoes. Body mass index (BMI) was calculated by dividing weight, in kilograms, by height, in meters, squared.

### 2.6 The dietary inflammatory index

Details regarding the development and validation of DII have been described elsewhere (Shivappa, Steck, Hurley, Hussey, & Hebert, 2014; Shivappa, Steck, Hurley, Hussey, Ma, et al., 2014). Briefly, the DII was developed based on literature search that was conducted to recognize the associations between inflammatory biomarkers and various food parameters, including several nutrients, foods, and bioactive compounds. The identification of these food parameters was not performed a priori; rather, they were identified prospectively, that is, dynamically as the search was conducted, in order to identify dietary components that influence the inflammation.

Of a possible 45 food parameters that comprise the full DII, the following dietary components were retrieved from the FFQ and used for DII calculation: carbohydrate, protein, fat, energy, alcohol, fiber, cholesterol, saturated fat, monounsaturated fat, polyunsaturated fat, omega‐3, omega‐6, trans‐fat, niacin, thiamin, riboflavin, vitamin B12, vitamin B6, iron, zinc, vitamin A, vitamin C, vitamin D, vitamin E, folic acid, beta carotene, magnesium, and selenium. The inability to account for all of the DII components is common in many studies that use FFQ for dietary assessment. The FFQ also provided information on onions and tea and the following bioactive compounds: flavan‐3‐ol, flavones, flavonols, and isoflavones.

The standard global mean value for each food parameter (which provides an estimate of a mean and standard deviation) was subtracted from each individual's dietary intake; afterward, the obtained value was divided by the standard deviation to create a z‐score. Subsequently, the *z*‐scores were converted to a proportion using the PROBNORM function in SAS (SAS Institute). These values were then centered by doubling the value and subtracting 1. This value was then multiplied by the inflammatory effect score for each food parameter. DII‐specific scores of all of the food parameter were then summed to create the overall DII score for every subject in the study. DII = b1 * *n*1 + b2 * *n*2…b(*n*) * *n*(*n*), where b refers to the literature‐derived inflammatory effect score for each food parameter; n refers to the food parameter‐specific‐centered percentiles, which were derived from the dietary data; and (*n*) refers to the total number of food parameters that will be available from this study. A more proinflammatory diet is indicated by a positive score, while a negative score reflects a diet that is more anti‐inflammatory. Finally, the DII scores were calculated per 1,000 calories/d consumed, which requires using an energy‐adjusted world referent database, and produced energy‐adjusted DII (E‐DII) scores.

### 2.7 Statistical analysis

Sample size was calculated based on the number of women diagnosed with BrCA in 2014 which was 1,174, 95% confidence interval, 5% margin of error, and 85% response distribution. The calculated sample size was 170. In general, about 20% of our population had been enrolled.

Differences in baseline variables among different tertiles were estimated using chi‐square tests for categorical variables and ANOVA for continuous variables. DII was categorized into tertiles, with cutpoints derived from data obtained in the controls. Logistic regression models were used to calculate odds ratios (ORs), and 95% CIs were estimated using, adjusting only for age first and then for age and energy in the second model. The third model was adjusted for additional adjustment including age, education, total energy, BMI, number of pregnancy, contraceptive use, lactation, smoking, and family history of BrCA. All tests were two‐sided, and the significance level was set at *p* < .05. Data were analyzed using SPSS^®^ for Windows version 23 (SPSS Inc.). Data were further stratified based on BMI values into overweight/obese (BMI ≥25 kg/m^2^) and nonobese (BMI ≤25 kg/m^2^); then, the associations between DII and BrCA were calculated using the aforementioned procedure.

## 3 RESULTS

One control participant dropped out from the study due to personal reasons. Thus, the final number of participants included in the statistical analyses was 199 controls and 200 cases. In this study, DII scores ranged from −4.08 (most anti‐inflammatory score) to 4.08 (most proinflammatory score). The general sociodemographic characteristics and lifestyle variables between cases and controls were presented elsewhere. General characteristics of case and control participants are presented in Tables 1 and 2, respectively. There were no statistical differences in sociodemographic factors and lifestyle habits across DII tertiles among cases; however, BMI and number of pregnancies factors of controls were statically different among DII tertiles.

**TABLE 1:** Participant characteristics by tertile of dietary inflammatory index among cases, Jordanian breast cancer case–control study (*n* = 200)

Variables	Tertile 1^a^ <−0.47	Tertile 2 −0.47 to −1.17	Tertile 3 >−1.17	*p*‐value^b^
Age (year)	48.3 ± 8.83	49.2 ± 8.60	49.0 ± 9.40	.842
BMI (kg/m^2^)	30.49 ± 5.56	28.9 ± 4.41	30.0 ± 5.46	.264
Smoking				
Yes	15	9	16	.283
No	44	55	61	
Marital status				
Married	45	49	60	.84
Single	10	7	9	
Divorced	1	4	3	
Widowed	3	4	5	
Education				
Illiterate	2	4	6	.366
Primary school	10	18	24	
High school	19	18	28	
Diploma	21	16	10	
Bachelor	3	5	6	
Higher education	4	3	3	
Work				
Yes	16	13	24	.345
No	43	51	53	
Physical activity (METs/wk)				
Inactive	51	60	70	.391
Minimally active	3	3	2	
HEPA active^c^	5	1	3	
Family with cancer				
Yes	32	35	42	.999
No	27	29	35	
Number of pregnancy				
0	13	10	12	.847
1–3	9	12	13	
4–6	23	26	28	
7–10	13	12	21	
>10	1	4	3	
Lactation				
Yes	38	42	50	.949
No	21	22	27	

Note

Values are mean ± *SD* or *n*.

Abbreviations: HEPA, Health‐Enhancing Physical Activity; METs, metabolic equivalents.

^a^ DII scores ranged from −4.08 to 4.08

^b^ Student's *t* test was used for continuous variables. Chi‐square test was used for categorical variables.

^c^ The Health‐enhancing physical activity category “active” included any participant who performed vigorous‐intensity activity on ≥3 d/wk, accumulated ≥1,500 MET‐min/wk, or who performed any combination of walking, moderate‐intensity or vigorous‐intensity activities ≥5 d achieving a minimum of ≥3,000 MET‐min/wk.

p‐value was set at < 0.05.

**TABLE 2:** Participant characteristics by tertile of dietary inflammatory index among control, Jordanian breast cancer case–control study (*n* = 199)

Variables	Tertile 1^a^ <−0.47	Tertile 2 −0.47 to −1.17	Tertile 3 >−1.17	*p*‐value^b^
Age (year)	48.8 ± 8.56	47.1 ± 7.90	46.4 ± 8.10	.225
BMI (kg/m^2^)	28.4 ± 5.26	29.2 ± 4.90	26.4 ± 4.69	**.004**
Smoking				
Yes	7	3	4	.341
No	58	63	64	
Marital status				
Married	52	49	55	.701
Single	8	11	10	
Divorced	1	1	2	
Widowed	4	5	1	
Education				
Illiterate	1	3	1	.209
Primary school	2	3	7	
High school	20	11	15	
Diploma	20	21	23	
Bachelor	15	21	19	
Higher education	7	7	3	
Work				
Yes	23	36	30	.142
No	42	30	38	
Physical activity (METs/wk)				
Inactive	52	52	58	.699
Minimally active	7	10	7	
HEPA active^c^	6	4	3	
Family with cancer				
Yes	25	27	21	.454
No	40	39	47	
Number of pregnancy				
0	9	13	22	**.010**
1–3	29	18	22	
4–6	20	23	16	
7–10	4	12	8	
>10	3	0	0	
Lactation				
Yes	47	44	39	.431
No	18	22	28	

Note

Values are mean ± *SD* or *n*.

Abbreviation: METs, metabolic equivalents.

^a^ DII scores ranged from −4.08 to 4.08

^b^ Student's *t* test was used for continuous variables. Chi‐square test was used for categorical variables. *p*‐value was set at <0.05.

^c^ The Health‐enhancing physical activity category “active” included any participant who performed vigorous‐intensity activity on ≥3 d/wk, accumulated ≥ 1,500 MET‐min/wk, or who performed any combination of walking, moderate‐intensity or vigorous‐intensity activities ≥5 days achieving a minimum of ≥3,000 MET‐min/wk.

The ORs (95% CIs) for the association between DII and BrCA are shown in Table 3. Results obtained from modeling DII scores a continuous variable in relation to the odds of developing BrCA showed no significant associations after adjusting for age (model 1), age and energy intake (model 2) or age, education, total energy intake, BMI, number of pregnancy, contraceptive use, lactation, smoking, and family history of BrCA (model 3). Tables 4 and 5 show the association between DII score and BrCA among normal weight and overweight/obese participants, respectively. Following the stratification of data based on the BMI values into overweight/obese (BMI ≥25 kg/m^2^) and normal weight (BMI <25 kg/m^2^), overweight/obese participants in the highest DII tertile had an OR of 1.77 (95% CI, 1.01–3.12) compared with participants in the lowest tertile, in the age‐adjusted models for DII tertiles. None of the other models for overweight/obese participants showed significant results.

**TABLE 3:** Odds ratios 95% confidence intervals for the association between dietary inflammatory index and breast cancer in a Jordanian case–control study (*N* = 399)

DII	Energy‐adjusted DII (tertiles^a^), OR (95% CI)			*p*_trend_ *	Energy‐adjusted DII (continuous) OR (95% CI)
	Tertile 1 (≤−0.47)	Tertile 2 (−0.47 to −1.17)	Tertile 3 (>−1.17)		
Age adjusted	1 (ref.)	1.08 (0.66–1.77)	1.27 (0.78–2.06)	.328	1.10 (0.94–1.25)
Age and energy adjusted	1 (ref.)	0.95 (0.57–1.59)	1.18 (0.72–1.95)	.508	1.03 (0.90–1.18)
Multivariate adjusted^b^	1 (ref.)	1.01 (0.55–1.84)	1.11 (0.61–2.01)	.753	1.00 (0.85–1.17)

Abbreviation: DII, dietary inflammatory index.

^a^ DII scores ranged from −4.08 to 4.08

^b^ Adjusted for age, education, total energy, body mass index, number of pregnancy, contraceptive use, lactation, smoking, and family history of breast cancer.

* *p*‐value for trend derived using the median approach.

**TABLE 4:** Odds ratios 95% confidence intervals for the association between dietary inflammatory index and breast cancer among nonobese participants in a Jordanian case–control study (*n* = 92)

DII	Energy‐adjusted DII (Tertiles^a^), OR (95% CI)			*p*_trend_ *
	Tertile 1 (≤−0.47)	Tertile 2 (−0.47 to −1.17)	Tertile 3 (>−1.17)	
Age adjusted	1 (ref.)	1.55 (0.47–5.11)	0.78 (0.27–2.31)	.518
Age and energy adjusted	1 (ref.)	1.29 (0.37–4.55)	0.70 (0.22–2.20)	.457
Multivariate adjusted^b^	1 (ref.)	1.47 (0.23–10.1)	0.19 (0.03–1.19)	.224

Abbreviation: DII, dietary inflammatory index.

^a^ DII scores ranged from −4.08 to 4.08

^b^ Adjusted for age, education, total energy, body mass index, number of pregnancy, contraceptive use, lactation, smoking, and family history of breast cancer.

* *p*‐value for trend derived using the median approach.

**TABLE 5:** Odds ratios 95% confidence intervals for the association between dietary inflammatory index and breast cancer among overweight and overweight/obese participants in a Jordanian case–control study (*n* = 307)

DII	Energy‐adjusted DII (tertiles^a^), OR (95% CI)			*p*_trend_ *
	Tertile 1 (≤−0.47)	Tertile 2 (−0.47 to −1.17)	Tertile 3 (>−1.17)	
Age adjusted	1 (ref.)	1.00 (0.58–1.73)	**1.77 (1.01–3.12)**	**.05** **0**
Age and energy adjusted	1 (ref.)	0.91 (0.52–1.59)	1.6 (0.9–2.86)	.121
Multivariate adjusted^b^	1 (ref.)	0.88 (0.46–1.70)	1.48 (0.74–2.97)	.324

Abbreviation: DII, dietary inflammatory index.

^a^ DII scores ranged from −4.08 to 4.08

^b^ Adjusted for age, education, total energy, body mass index, number of pregnancy, contraceptive use, lactation, smoking, and family history of breast cancer.

* *p*‐value for trend derived using the median approach and it was set at <0.05.

## 4 DISCUSSION

Cancer, a global leading cause of death, appears often to be related to chronic, low‐grade inflammatory state that can be triggered by a microenvironment such as diet (Phillips et al., 2019). However, in this Jordanian case–control study the consumption of proinflammatory diet was not associated with an increased BrCA except among overweight/obese women.

The association between DII and cancer risk was recognized for various other types of cancer including prostate, colorectal, and pancreatic; nevertheless, the evidence was not conclusive regarding BrCA (Hoang et al., 2019; Shivappa, Blair, et al., 2017; Shivappa et al., 2016; Tabung et al., 2015). Several modifiable and non‐modifiable factors have been identified for their association with BrCA risk, including genetics, age, reproductive history, hormone use, diet, alcohol consumption, smoking, physical activity level, and obesity (McKenzie et al., 2015; Shivappa et al., 2015). In a large prospective cohort, a combination of healthy behaviors (never smoke, no alcohol consumption, high level of physical activity, being normal weight, and having a healthy diet) was found to have an inverse association with BrCA risk compared with unhealthy lifestyle as measured by healthy lifestyle index score (McKenzie et al., 2015). In their study, McKenzie et al. (2015) detected that a one‐point increment in the healthy lifestyle index score corresponded to a 3% reduction in BrCA risk, which highlights the contribution of modifiable factors on the pathogenesis of BrCA. Although the results of the current study have been adjusted for several confounders, the association between DII and BrCA risk did not reach the level of significance for the study as a whole. In accordance with our results, several previous observational studies and clinical trials have failed to link BrCA risk with dietary quality (Fung et al., 2005; Prentice et al., 2006; Smith‐Warner et al., 2001; Terry et al., 2001). The multifactorial etiology of BrCA, as well as the complexity of the diet, could contribute to the controversy regarding the association between BrCA and dietary factors due to the difficulty to nullify the influence of all of these factors.

Shivappa et al. (2016) detected 11% increase in the hazard ratio (95% CI: 1.00–1.22) for BrCA in association with proinflammatory diet as evidenced by higher DII, and the association was stronger in obese women (HR = 1.35; 95% CI: 1.10–1.66) (Shivappa, Blair, et al., 2017). Additionally, for obese women, a 5% higher risk was associated with a one‐unit increase in DII score (95% CI: 1.02–1.12). Similarly, other studies supported such association (Shivappa, Hebert, et al., 2017; Shivappa et al., 2015). On the other hand, in accordance with the results of our study, no association was discovered between the incidence of BrCA and DII among two different populations of postmenopausal women (Ge et al., 2015; Tabung et al., 2016). More research is warranted to examine the inflammatory potential of diet in a larger sample to test the effects on BrCA incidence. In this study, higher DII scores (third tertile compared with the reference tertile) were associated with 77% increases in the risk of developing BrCA in participants with BMI ≥25. Although this association was at borderline significance (95% CI: 1.01 – 3.12 and *p*‐trend .05), such association did not appear in normal weight individuals (BMI <25 kg/m^2^). This finding is compatible with earlier‐mentioned studies and points to the role of adiposity in BrCA risk, possibly via the consumption of a proinflammatory diet.

This is one of the few studies that have examined the association between DII and BrCA risk in Middle East region (Jalali et al., 2018; Vahid et al., 2018). Despite its strengths, this study has several limitations. First, we cannot exclude selection bias. Second, information bias always is of concern in a case–control study. Third, this study is relatively small compared with the very large sample sizes that have been used in other DII‐focused studies (Accardi et al., 2019; Ge et al., 2015; Tabung et al., 2016). However, the calculated sample size was representative considering the incidence rate of BrCA in both sexes that had been reported by Jordanian Cancer Registry in 2014 (Ministry of Health, N.‐C. D. D., 2014). Fourth, we were not able to account for all 45 components of the original DII. In this population, missing information included food parameters such as garlic and onion—and these may have played a role in this association. However, the absence of several food parameters such as turmeric and thyme is not of great concern because of the infrequency of consumption.

Although the relationship between the proinflammatory potential of the diet and BrCA risk is yet to be clarified, maintaining higher diet quality and adherence to dietary recommendations for balanced, healthy diet would boost health and reduce the risk for chronic diseases.

## CONFLICT OF INTEREST

SSH, RM, LM, and RFT declare that they have no conflict of interests. Disclosure: Dr. James R. Hébert owns controlling interest in Connecting Health Innovations LLC (CHI), a company that has licensed the right to his invention of the DII from the University of South Carolina in order to develop computer and smartphone applications for patient counseling and dietary intervention in clinical settings. Dr. Nitin Shivappa is an employee of CHI.

## ETHICAL APPROVAL

The study was conducted in accordance with the ethical standards of the responsible committee on human experimentation and with the Helsinki Declaration of 1975, as revised in the proposal was approved by the Institutional Review Board of the 2 hospitals: King Hussein Cancer Center (KHCC) and Al‐Basheer (16 KHCC 57 and 2250, respectively).
